# A three-tier framework for monitoring antiretroviral therapy in high HIV burden settings

**DOI:** 10.7448/IAS.17.1.18908

**Published:** 2014-04-28

**Authors:** Meg Osler, Katherine Hilderbrand, Claudine Hennessey, Juanita Arendse, Eric Goemaere, Nathan Ford, Andrew Boulle

**Affiliations:** 1 Centre for Infectious Diseases, Epidemiology and Research, School of Public Health and Family Medicine, University of Cape Town, Cape Town, South Africa; 2 Médecins Sans Frontières, Southern African Medical Unit, Cape Town, South Africa; 3 Department of Health, Provincial Government of the Western Cape, Cape Town, South Africa

**Keywords:** monitoring, antiretroviral therapy, TIER.Net, HIV, electronic register, three-tier system, eKapa

## Abstract

The provision of antiretroviral therapy (ART) in low and middle-income countries is a chronic disease intervention of unprecedented magnitude and is the dominant health systems challenge for high-burden countries, many of which rank among the poorest in the world. Substantial external investment, together with the requirement for service evolution to adapt to changing needs, including the constant shift to earlier ART initiation, makes outcome monitoring and reporting particularly important. However, there is growing concern at the inability of many high-burden countries to report on the outcomes of patients who have been in care for various durations, or even the number of patients in care at a particular point in time. In many instances, countries can only report on the number of patients ever started on ART. Despite paper register systems coming under increasing strain, the evolution from paper directly to complex electronic medical record solutions is not viable in many contexts. Implementing a bridging solution, such as a simple offline electronic version of the paper register, can be a pragmatic alternative. This paper describes and recommends a three-tiered monitoring approach in low- and middle-income countries based on the experience implementing such a system in the Western Cape province of South Africa. A three-tier approach allows Ministries of Health to strategically implement one of the tiers in each facility offering ART services. Each tier produces the same nationally required monthly enrolment and quarterly cohort reports so that outputs from the three tiers can be aggregated into a single database at any level of the health system. The choice of tier is based on context and resources at the time of implementation. As resources and infrastructure improve, more facilities will transition to the next highest and more technologically sophisticated tier. Implementing a three-tier monitoring system at country level for pre-antiretroviral wellness, ART, tuberculosis and mother and child health services can be an efficient approach to ensuring system-wide harmonization and accurate monitoring of services, including long term retention in care, during the scale-up of electronic monitoring solutions.

## Introduction

The provision of antiretroviral therapy (ART) in low- and middle-income countries is a chronic disease intervention of unprecedented magnitude [1–3] and is the dominant health systems challenge for countries with a high burden of HIV infection, many of which rank among the poorest countries in the world. ART provision is characterized by its scale; the accumulation of numbers on treatment, given the requirement for lifelong therapy and the associated burden on health services; and national and international funding [4].

Substantial external investment, together with the requirement for service evolution to adapt to changing needs, including the constant shift to earlier ART initiation [1, 5, 6], makes outcome monitoring and reporting particularly important. However, there is growing concern at the inability of many high-burden countries to report on the outcomes of patients who have been in care for various durations, or even the number of patients in care at a particular point in time [4, 7–10]. In many instances, countries can only report on the number of patients ever initiated on ART.

For chronic disease care, the preferred means of monitoring progress is through cohort monitoring, which follows groups of patients over time and reports on key baseline and outcome variables [11, 12], which in the case of HIV care may include immunological, clinical and virological indicators [13, 14]. Typically, data are aggregated per cohort at standard treatment durations measured from the start of care.

Monitoring of primary care interventions has often been based on tallying the number of services rendered to inform the allocation of resources, with pervasive concerns on the reliability of these data [15]. Against this backdrop, there are many reasons for the failure to establish and maintain robust country-level HIV cohort monitoring systems in many high-burden countries. Chief amongst these are the rapid scale-up of ART, limited human and monetary investment in monitoring, and limited appreciation of the value of cohort monitoring to inform both policy and facility management.

In addition, monitoring may be done by different actors and services with poor coordination between sites. In many settings, ART provision has involved a collaborative effort by many actors, including the public and private sector, national and international nongovernmental organizations (NGOs), academic research groups and external donors. Varied and complex electronic medical record (EMR) systems have been created either with particular research interests in mind or in order to fulfil parallel reporting requirements stipulated by donor agencies [9, 16]. Most of these tools are not, however, simple or robust enough for use at scale, and depend on facility-based infrastructure, network capacity and stability, leaving individual treatment sites and health authorities without viable standardized tools to monitor the ART programme.

There have been notable exceptions in terms of country level or regional reporting. The government of Malawi has an impressive track record of peer-reviewed outputs based on their national monitoring system for ART [17–20]. The Western Cape province of South Africa, on a smaller scale, has maintained cohort reporting for over 10 years during which time (by October 2013) over 175,000 patients have been initiated on ART for the first time, across 227 facilities. The common themes for both programmes have been a single monitoring system, strong system stewardship and a phased evolution, which began with paper-based systems that then guided the careful development of electronic data capture [14].

In this paper we describe a pragmatic, multi-tier and fully interoperable technology mix that limits dependencies and points of system failure, offering a viable and context-appropriate framework for ART monitoring in resource-constrained settings. This three-tier approach was developed in the Western Cape of South Africa, which has experienced rapid scale-up of ART over the last decade (see case study at the end of the manuscript) [14, 21, 22]. Paper-based registers, electronic registers and EMR solutions are combined in a unified system to produce common nationally required indicators, and rapid migration options between tiers as resources or monitoring needs change.

## Rationale for a multi-tier solution

Over the past decade of ART scale-up, many countries have recognized the need to transition from paper to EMR systems in order to manage ever increasing patient numbers. This transition has been mainly driven by high patient burden or the length of follow-up where extracting data from patient records or aggregating paper registers have become unwieldy and unsustainable. One study in Malawi reported that high-burden clinics managing in excess of 2000 patients on ART need up to five days to extract quarterly cohort reports directly from patient records, with facilities sometimes obliged to halt services during this period, despite the support of a team from the National AIDS Office [23]. Implementing a collection tool such as a paper ART register can make extracting routine quarterly reports less burdensome. Nonetheless, with time a multitude of registers accumulate and patient follow-up duration exceeds what the registers were designed for, making the storage, recording and aggregation of data more cumbersome.

Despite paper register systems coming under increasing strain, the evolution from paper directly to an EMR solution is not viable in many contexts. Most EMR systems require wide area networks, facility-level infrastructure including computers and local networks, and structured helpdesk support. Well-designed and context-appropriate systems might still fail due to their dependency on infrastructure and support.

**Figure 1:**
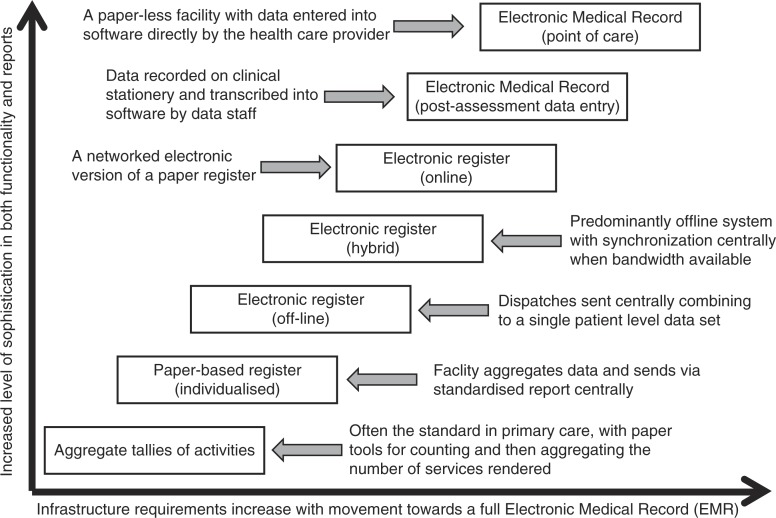
Different candidate tiers of a multi-tier monitoring system.

Transitioning from paper directly to EMR systems is often not immediately feasible, and implementing a bridging solution can be a pragmatic alternative. These middle tier or bridging solutions include electronic implementation of the paper registers through offline or online solutions, or a hybrid of the two. Figure 1 illustrates the potential increments in sophistication in disease-monitoring solutions, which are candidate tiers in a multi-tier implementation.

It is not practical to implement and support all potential levels depicted in Figure 1. Implementing one middle tier can, however, make the evolution from paper to EMR solutions more viable. Digitization can happen more rapidly via direct back-capture from the paper registers already in place, whilst the infrastructure requirements and overheads are minimal in comparison to networked EMR solutions. A simple offline electronic version of the paper register has relatively few barriers to installation, only requiring a computer and a stable power source.

A three-tiered monitoring approach allows Ministries of Health to strategically implement one of the tiers in each facility offering HIV and/or ART services. The choice of tier is based on context and resources at the time of implementation; as resources and infrastructure improve, more facilities will transitionto the next highest and more technologically sophisticated tier.

**Figure 2:**
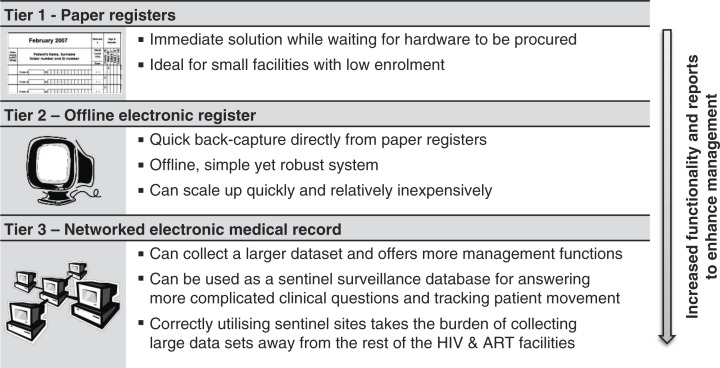
A three-tier monitoring and evaluation system capable of working together in a health region (one choice per facility) to ensure all contexts have an appropriate and viable way to monitor care across all levels of the health services.

In South Africa, the three-tier monitoring and evaluation system for ART (Figure 2) was adopted by the National Department of Health in December 2010. The three-tier monitoring and evaluation system allows for data to be reported centrally, but has the patient and programme management by facility as its main focus. As a facility moves up in tiers, the number of reports to improve facility management of the health service increases. These reports are required to be used locally and are mandated and supported by national standard operating procedures for the monitoring of ART. In addition to the monthly patient totals and quarterly cohort reports, the paper-based system can be used to extract missed appointment lists for tracing patients via community health workers; the middle-tier offline system automates the generation of missed appointment and defaulter lists, can generate staff work load reports (for burden, accountability and recognition) and missing laboratory result reports amongst others; the third-tier or networked EMR in addition allows for centralized data quality control and has more built-in clinical validations.

## Key principles of a three-tier solution

### Each tier produces the same minimum set of reports

**Figure 3:**
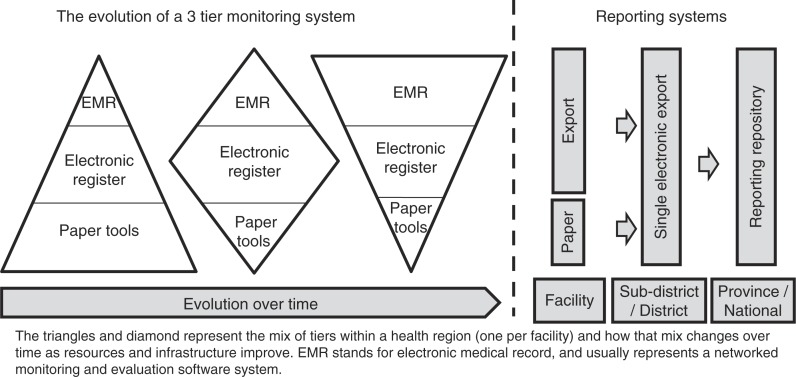
Evolution from paper systems to full EMR systems over time.

Each tier should produce the same nationally required monthly enrolment and quarterly cohort reports so that outputs from the three tiers can be aggregated into a single database at any level of the health system, giving programme managers a better understanding of the burden of care, equity of access, quality of service, retention in care and other outcomes of the programme (Figure 3).

Although each tier produces the same monthly and quarterly cohort reports, reports and modules to facilitate service management at a facility or district improve as facilities migrate to the next tier. For example, an offline computerized implementation of the registers can provide listings of patients who have missed appointments, defaulted, transferred out, are on second-line medication or are clinically eligible for but not yet on ART. EMR solutions typically offer more comprehensive functionality in addition to these reports, such as detailed appointment systems, workload management tools, access to laboratory, pharmacy and vital status data, and closer tracking of patient movement through the system.

### Standardization and interoperability

An import/export data exchange standard (DES) enables the transfer of data from one software system to another. In addition to allowing interoperability between tiers, a DES can provide an interoperability solution in countries that have a multitude of existing software applications of similar functionality, which evolved organically, ensuring data from each application is imported into a single national data set. Criteria such as DES compliance and validated nationally required reports can then be set by governments as prerequisites for the continued sanctioning of software solutions. Multiple solutions can in some instances create healthy competition and reduce risk of failure by preventing dependence on a single system, which might not scale well or fail to meet future functionality requirements, or for which contractual complications may be encountered.

The DES for ART in South Africa has been based on the HIV Cohorts Data Exchange Protocol [24], which is well-documented and further facilitates rapid analyses of exported data to address queries that are not catered for by routine reports.

### Using a middle tier as a migration strategy towards an EMR

A DES also provides a “stepping stone” for expedited back-capture of patient histories directly from paper ART registers without the extra burden of finding, drawing, re-capturing and re-filing every physical patient folder. In our experience in South Africa, back capturing data for a patient who started ART in 2004 from a paper register into a middle-tier system takes 2 to 3 minutes in comparison with 5 to 20 minutes if capturing from a patient folder depending on the location of the patient files and whether or not standard clinical stationery was used by clinicians. Once a site is fully back-captured, a DES export can be created for direct import into EMR software if resources and management capacity warrant it.

## Practical considerations in determining the optimal alignment of solutions

Paper-based systems are likely to remain used in facilities for years to come, especially in small rural clinics and those without stable electricity, as well as new ART services. The opening of a new ART service should not be delayed due to procurement of hardware or cabling, and paper systems allow for immediate implementation of a monitoring system with rapid migration to an electronic register as resources become available.

### Choice of tier does not reduce data staff requirements or necessarily impact on data quality

Regardless of tier, dedicated staff time is required to transcribe patient data on a daily basis and to extract and compile required data for monthly, quarterly and ad hoc reports. Except in very small services, dedicated data staff are essential irrespective of tier, although depending on clinic size they may also have other administrative responsibilities at busier times of day. The key function of abstracting specific parameters from the structured clinical record of each patient seen each day and then capturing this in a paper register or electronic system cuts across solutions. The integrity of this process in terms of source clinical record-keeping, completeness of folder processing and accuracy of capture are what ultimately determine the quality of data, rather than the choice of system into which data are captured. Managing the integrity of reports is, however, easier with electronic solutions, although errors in manually extracted reports can be readily identified through rule-based consistency checks on the aggregate data.

### Not all sites need to have an EMR at the outset

Smaller services with low monthly enrolment may not need to move to an electronic system; however, larger more mature sites with multiple registers could benefit substantially from appropriate electronic solutions. Ministries of Health should also consider establishing a small number of sentinel sites during the early stages of digitization that are supported by implementing partners or academic groups that collect a larger set of more closely interrogated data. These few sentinel sites can help to answer questions raised by a Ministry regarding clinical or operational issues without burdening all facilities in the country with collection of an expanded data set [14].

### A successful lower tier as a prerequisite to moving to the next tier

With each higher tier comes added complexity and the need for additional support. The second-tier solutions require computer literate staff, computer availability, and software and hardware support, in addition to the training and protected staff time that are common requirements of all tiers. Third-tier solutions additionally require central database and system control and network maintenance. Therefore, if a lower tier is not successfully working (given certain workload parameters), moving to a higher tier is unlikely to be successful. Digitization of registers into a tier-2 solution that resembles the tier-1 paper registers is a far more efficient digitization strategy than comprehensive back-capture of physical patient folders into an EMR system.

### Ensuring ability to benefit from added functionality of EMR systems

Ultimately, tier-3 solutions can offer additional functionality beyond routine monitoring and patient listings, including linking to laboratory or pharmacy data and software systems. To benefit from the added functionality requires availability of additional infrastructure (networking and hardware at more service points), staff and management capacity. In particular, support systems need to be in place and reliable so that faulty equipment is rapidly replaced, network bandwidth is sufficient and network stability is guaranteed.

Extensive support for clinical governance is potentially available from high-end EMR systems, including real-time or asynchronous decision support and tools to improve service efficiency. Both require committed clinical and management champions who will use these tools to iteratively improve services. Some of the largest sites with the greatest potential to benefit from EMR systems are also the most challenging sites to ensure stability of infrastructure, staff and management processes.

### System-wide uniformity may trump facility considerations

Early stages of evolution to an electronic monitoring platform may be driven by resources, patient burden and equity considerations. However, if a country has finished transitioning to a multi-tier system with the majority of sites on tiers 2 or 3, it does not make sense to continue with paper registers at the smallest facilities. Electronic monitoring systems can provide patient-level data to higher levels, which brings additional programme knowledge when combined with other health data (in comparison to aggregate numbers from paper systems). Similarly, if networks are in place and most sites are already using a tier-2 system, it may make sense to further scale-up tier-3 solutions. The true benefits of an EMR will only be realized once a large area is fully utilizing a single networked solution. Patient movements can be comprehensively tracked, patient histories are available to referral or emergency centres, and laboratory data and most recent clinic visits and outcomes can be linked or imported.

## Consolidating an electronic register platform across priority programmes

While middle-tier electronic registers are proving to be a rapidly scalable solution for HIV and ART monitoring in some settings, including in South Africa as part of the three-tier approach, another example where electronic registers have been widely implemented is for monitoring tuberculosis control. ETR.Net™ (Electronic Tuberculosis Register) has been implemented in eight countries and collects and reports on demographic, case finding and outcome data for patients receiving tuberculosis treatment. This simple offline middle-tier approach could also provide important benefits to services such as maternal and child health services (MCH) in developing countries, where longitudinal outcome data are necessary, but rolling out EMR software to all facilities offering these services would be too expensive and resource intensive; services for the prevention of mother-to-child transmission (PMTCT) of HIV, another global health priority, could also benefit from such an approach. With the WHO 2013 guidelines for ART now promoting the integrated provision of ART through TB, antenatal care and MCH sites, the need for such platforms across different clinic settings will become increasingly important [25]. Using a tiered platform across HIV, TB and MCH, health services will have a common data platform for outcome reporting of priority programmes for use at facility level. It could provide service managers with a better understanding of co-infection rates and multiple health services access, along with improved tools for targeting interventions.

There are many overlapping challenges in providing robust programme monitoring and treatment provision for HIV/ART, tuberculosis and MCH services. In addition, patients may attend more than one service at any given time. Deploying a middle-tier software solution incorporating these three linked priority disease programmes could be the catalyst to natural service integration at facility level including reception staff, data staff, counsellors and clinicians. An integrated priority disease approach to these three dynamic and rapidly changing service domains requiring outcome reporting would maintain focus on these diseases and at the same time slowly merge the vertical and separate monitoring approaches that were initially taken.

## Conclusions

Implementing a three-tier monitoring and evaluation system at country level for HIV and ART services can be an efficient approach to ensuring system-wide harmonization and accurate monitoring of services, including long-term retention in care. The different tiers allow for rapid roll out of services while maintaining a single national data set that includes data from all sites regardless of size or context. The inclusion of a uniform DES in the second and third tier gives added flexibility, allowing different software solutions to coexist and for rapid migration between tiers. The middle-tier electronic register assists with rapid digitization of paper treatment registers and can facilitate eventual migration to EMR systems through the uniform DES. The second and third tiers provide added management reports and additional functionality and can provide the platforms required for integrated monitoring and evaluation of TB, MCH and PMTCT services. EMR systems already in place or implemented in sites meeting country-stipulated criteria can play a vital role initially in providing more detailed data from sentinel sites and over time may evolve into more comprehensive solutions when resources and infrastructure allow facility evolution to networked EMR systems.

## Case study

South Africa rolled out a free ART service in the public health sector beginning in April 2004 [21]. Through close collaboration with academic centres and donor agencies, the Western Cape started the same free services from May of 2001 [22]. The Western Cape has an estimated population of just under 6 million people and is the fourth most populous of the South Africa's nine provinces. By mid-2012, the Actuarial Society of Southern Africa HIV model projections estimated that 260,000 adults were living with HIV in the Western Cape Province and there would be an estimated 13,000 new infections in the 2012–2013 financial year. At the end of December 2013, a reported 150,000 people remained in care within the ART services. Monthly reporting is from all public health facilities offering ART services.

The Western Cape monitoring and evaluation programme for ART services started as a combination of paper registers at the facilities scaling up ART services and EMR software called EKAPA (Evaluation of the Khayelistsha AIDS ProgrAm) at the initial Khayelitsha sentinel sites. This two-tier system, with the majority of sites using paper-based registers, was successfully monitoring outcomes for the entire cohort up to 2008, during a period when the programme was still young and enrolment was relatively low [14]. The paper registers allowed patients to be followed for up to four years on treatment within a single register. It was envisaged that by the time the first patients completed the register, electronic solutions would be in place. A number of delays in the provision of adequate networking infrastructure, computer hardware and software development (EKAPA was being redeveloped as a Provincial system), together with the constant programme expansion, resulted in a failure to migrate sites onto an electronic system as originally envisaged. In spite of creative adaptations of the paper-based system to cope with ever increasing patient numbers and durations on ART, clerical staff in large or mature sites began to experience increasing strain maintaining the paper-based registers and extracting monthly and quarterly cohort reports.

A stand-alone electronic HIV register had been developed by the University of Cape Town Centre for Infectious Disease Epidemiology and Research as a potential digitization option for paper registers, and in line with WHO guidance was intended to eventually encompass multiple priority HIV-linked interventions, which required outcome reporting (HIV treatment, tuberculosis treatment and mother and child health services incorporating the prevention of mother-to-child transmission of HIV). This application (TIER.Net for the Three Interlinked Electronic Registers) became the middle tier of the three-tier monitoring and evaluation system and enabled the rapid digitization of the paper registers, in many cases obviating the need to go back to source folders.

The quality of data improved when migrating from paper to electronic solutions, particularly in the reporting of patients lost to care where large sites using paper registers struggled to identify and account for all losses distributed across multiple registers. In addition to improving accuracy, the auto-calculation of the cohort reports after migration to electronic registers overcame the need to reserve dedicated clerical time to extract reports, thereby improving the timeliness of reporting.

Table 1 reflects routine cohort data from the Western Cape Antiretroviral programme with follow-up data to the 31 December 2013. Information reflects patients newly initiated on treatment and is based on a combination of reports from paper antiretroviral registers, TIER.Net (the offline tier-2 software) and EKAPA (the tier-3 networked EMR). These data were predominantly collected by facilities using TIER.Net (198 of 227 facilities), but a substantial proportion (20%) of patients were followed by the larger sites using EKAPA; only a few sites remained on paper-based systems. Just over 87% of the facilities offering ART reported cohort outcomes for inclusion in the presented data.

**Table 1:** Western Cape ART programme reporting from routine monitoring and evaluation systems

	Year	2001	2002	2003	2004	2005	2006	2007	2008	2009	2010	2011	2012	Total
Baseline information	Number starting ART (naive)	88	308	596	2811	5637	8140	9606	15,069	19,018	23,915	26,732	31,014	142,934
	Male (%)	27.7	30.7	29.7	31.1	32.3	34.4	36.0	34.3	35.9	34.9	35.6	35.6	35.1
	Paediatric (%)	5.7	31.2	27.0	10.6	8.2	7.8	6.1	5.1	4.5	4.0	3.1	2.6	4.5
	ART experienced (%)	7.4	1.9	2.1	3.8	3.4	3.4	4.2	5.2	6.0	5.8	5.5	4.2	5.0
	CD4<100 cells/µl (%)	78.6	72.5	61.3	51.2	45.4	43.1	40.2	34.8	35.1	29.8	23.8	18.9	30.2
	CD4≥200 cells/µl (%)	0.0	5.2	6.5	6.2	6.7	8.9	12.0	16.3	18.1	28.2	38.5	50.2	28.8
ART status after one year on ART	Remaining in care (%)	85.1	87.9	89.7	88.0	87.2	86.2	84.9	86.4	83.7	81.1	77.0	–	82.7
	LTF (cumulative %)	0.0	2.0	1.7	5.1	6.2	8.3	9.9	10.0	13.0	15.6	20.0	–	13.4
	Mortality (cumulative %)	14.9	10.2	8.6	7.0	6.5	5.5	5.3	3.6	3.4	3.2	3.1	–	4.0
	Second line (%)	0.0	0.0	0.8	0.9	0.7	0.8	0.6	0.9	0.9	1.2	1.3	–	1.0
	Viral load suppression (%)	82.4	74.4	87.0	87.3	89.0	87.7	88.7	87.9	85.8	85.0	87.0	–	86.8
	Viral load completion (%)	91.9	74.3	66.7	76.8	81.3	76.9	72.0	71.3	67.4	67.0	59.5	–	68.5
ART status after four years on ART	Remaining in care (%)	76.5	79.4	75.6	73.5	72.1	70.1	67.5	64.5	–	–	–	–	68.3
	LTF (cumulative %)	1.2	5.5	10.5	14.2	16.3	20.3	23.2	28.4	–	–	–	–	22.3
	Mortality (cumulative %)	22.4	15.1	13.9	12.2	11.6	9.6	9.3	7.1	–	–	–	–	9.3
	Second line (%)	10.8	16.5	12.2	9.3	8.1	8.6	9.3	10.0	–	–	–	–	9.3
	Viral load suppression (%)	87.1	82.7	89.9	89.7	88.5	82.2	84.6	84.6	–	–	–	–	85.2
	Viral load completion (%)	95.4	70.1	66.5	74.6	78.7	74.9	72.6	62.1	–	–	–	–	70.8
ART status after eight years on ART	Remaining in care (%)	66.3	64.7	60.8	56.6	–	–	–	–	–	–	–	–	58.3
	LTF (cumulative %)	8.8	14.3	21.6	26.6	–	–	–	–	–	–	–	–	24.2
	Mortality (cumulative %)	25.0	21.1	17.5	16.8	–	–	–	–	–	–	–	–	17.6
	Second line (%)	20.8	28.5	14.2	16.3	–	–	–	–	–	–	–	–	17.3
	Viral load suppression (%)	100.0	82.5	87.4	89.5	–	–	–	–	–	–	–	–	89.1
	Viral load completion (%)	64.2	36.6	59.3	68.0	–	–	–	–	–	–	–	–	63.2

ART: antiretroviral therapy; LTF: lost to follow-up.

The table describes the baseline characteristics, clinical status and outcomes of yearly cohorts (columns) per duration of time on ART (rows). Each grouped set of rows reports on indicators per duration on ART for that particular cohort, with duration or time on ART increasing as one scrolls down the column. When the programme first started in 2001, over 78% of those initiating ART had a CD4 count<100 cells/µl. This proportion dropped over time, with only 19% initiating ART with such low CD4 counts in 2013. Mortality during the first year on ART (especially the first three months) has dropped over time and is correlated with the increase in CD4 counts at ART initiation within each cohort. Retention (number remaining in care at the same facility where they initiated ART) at 12 months was greater than 85% until 2009; however, with each year this figure decreased slightly. The drop in retention over time is related to the ever increasing number of patients enrolling in care. As the programme grows, the proportion of people lost to ART care increases, and this has forced policy makers and innovators to think about changes to the traditional models of care in order to decongest the ART services.
